# Very Long-Term Prognostic Role of Admission BNP in Non-ST Segment Elevation Acute Coronary Syndrome

**DOI:** 10.5935/abc.20160021

**Published:** 2016-03

**Authors:** Fernando Bassan, Roberto Bassan, Roberto Esporcatte, Braulio Santos, Bernardo Tura

**Affiliations:** 1 Universidade do Estado do Rio de Janeiro, Rio de Janeiro, RJ - Brazil; 2 Departamento de Coronariopatia - Instituto Nacional de Cardiologia, Rio de Janeiro, RJ - Brazil; 3 Pontíficia Universidade Católica do Rio de Janeiro, Rio de Janeiro, RJ - Brazil; 4 Hospital Pró-Cardíaco, Rio de Janeiro, RJ - Brazil; 5 Departamento de Pesquisa Clínica - Instituto Nacional de Cardiologia, Rio de Janeiro, RJ - Brazil

**Keywords:** Natriuretic Peptide, B-Type / mortality, Prognosis, Acute Coronary Syndrome, Myocardial Ischemia

## Abstract

**Background:**

BNP has been extensively evaluated to determine short- and intermediate-term prognosis in patients with acute coronary syndrome, but its role in long-term mortality is not known.

**Objective:**

To determine the very long-term prognostic role of B-type natriuretic peptide (BNP) for all-cause mortality in patients with non-ST segment elevation acute coronary syndrome (NSTEACS).

**Methods:**

A cohort of 224 consecutive patients with NSTEACS, prospectively seen in the Emergency Department, had BNP measured on arrival to establish prognosis, and underwent a median 9.34-year follow-up for all-cause mortality.

**Results:**

Unstable angina was diagnosed in 52.2%, and non-ST segment elevation myocardial infarction, in 47.8%. Median admission BNP was 81.9 pg/mL (IQ range = 22.2; 225) and mortality rate was correlated with increasing BNP quartiles: 14.3; 16.1; 48.2; and 73.2% (p < 0.0001). ROC curve disclosed 100 pg/mL as the best BNP cut-off value for mortality prediction (area under the curve = 0.789, 95% CI= 0.723-0.854), being a strong predictor of late mortality: BNP < 100 = 17.3% vs. BNP ≥ 100 = 65.0%, RR = 3.76 (95% CI = 2.49-5.63, p < 0.001). On logistic regression analysis, age >72 years (OR = 3.79, 95% CI = 1.62-8.86, p = 0.002), BNP ≥ 100 pg/mL (OR = 6.24, 95% CI = 2.95-13.23, p < 0.001) and estimated glomerular filtration rate (OR = 0.98, 95% CI = 0.97-0.99, p = 0.049) were independent late-mortality predictors.

**Conclusions:**

BNP measured at hospital admission in patients with NSTEACS is a strong, independent predictor of very long-term all-cause mortality. This study allows raising the hypothesis that BNP should be measured in all patients with NSTEACS at the index event for long-term risk stratification.

## Introduction

In spite of the large knowledge gathered in the last few decades in identifying clinical and laboratory variables to determine short- and intermediate-term prognosis in patients with acute coronary syndrome (ACS),^1-5^ their long-term prediction capability remains mostly unknown. B-type natriuretic peptides (BNP) are one of these markers and have been extensively evaluated for this purpose with very good performance,^6-10^ but no study has dealt with their value in correctly identifying those individuals at high risk of death in the very long-term.

We had the opportunity to prospectively, systematically collect clinical and laboratory data in a cohort of patients examined in the Chest Pain Unit of an Emergency Department, to whom a comprehensive diagnostic and risk-stratification protocol was used.^11^ Several clinical and laboratory markers were evaluated upon admission to establish the diagnosis of ACS. Patients were then treated accordingly and followed up to determine their long-term natural history. The aim of this study was to determine the prognostic role of the admission BNP level in the 10-year all-cause mortality of those individuals who had a final diagnosis of non-ST segment elevation ACS (NSTEACS).

## Methods

### Study population and data collection

We prospectively studied 723 consecutive patients seen in the Emergency Department of the Pró-Cardíaco Hospital, a private, tertiary, cardiology-oriented institution in Rio de Janeiro, Brazil, between January 1^st^, 2002, and December 31^st^, 2003. They complained of chest pain or discomfort in the preceding 12 hours due to possible acute cardiac ischemia, and their admission electrocardiogram (ECG) showed no ST-segment elevation. These patients were routinely managed in the Chest Pain Unit with a systematic diagnostic protocol recommending the following: 1) cardiac markers: admission and serial (every 3 hours) creatine kinase-MB mass (CKMB) and/or troponin-I levels; 2) admission and serial ECG; 3) echocardiogram (in many patients); 4) if neither myocardial necrosis nor rest ischemia were detected, a stress test (either treadmill ECG or single-photon emission computed tomographic myocardial scintigraphy). Coronary angiography and revascularization procedure were performed at the discretion of the attending physician. TIMI risk score was calculated in all patients fulfilling the diagnosis of ACS. Based on the above work-up, 237 patients received a final diagnosis of NSTEACS, constituting the initial study population. Of these, 13 individuals were lost sometime during the 10-year follow-up. The remaining 224 patients represent the final study sample.

Patients with suspected (intermediate to high pretest probability) or straightforward admitting diagnosis of life-threatening disorders other than ACS, such as aortic dissection and pulmonary embolism, were also immediately submitted to appropriate imaging tests and, if confirmed, diverted from the above mentioned diagnostic pathway. These patients were excluded from this study.

For this prospective study, plasma BNP was incorporated into the diagnostic protocol and obtained on admission with the purpose of correlating BNP with the final discharge diagnosis and follow-up prognosis. BNP results were available to treating physicians. This study complies with the Declaration of Helsinki and its protocol was approved by the hospital ethics committee. All patients provided written informed consent.

### Biochemical analysis

Plasma BNP was immediately analyzed on the same EDTA-anticoagulated blood sample collected on admission for troponin-I, using the quantitative immunofluorescence assay (Biosite, California, USA). The analytic sensitivity of the assay is less than 5 pg/mL and the upper normal limit is considered to be 100 pg/mL. Plasma troponin-I was measured by immunofluorescence assay (Dade-Behring, Marburg, Germany). The analytic sensitivity of the assay is 0.1 ng/mL and any value above this was considered diagnostic of non-ST-segment elevation myocardial infarction (NSTEMI) in this study.^12,13^ The coefficient of variation of the 99th percentile of the diagnostic value of the assay (0.1 ng/mL) is 10%.

### Clinical data and diagnosis on hospital discharge

All demographic and clinical data were prospectively obtained on hospital admission and during Emergency Department and hospital stay until final diagnosis was reached. NSTEMI was diagnosed when a troponin-I level above 0.1 ng/mL in any sample was found during the first 9 post-admission hours with a rise and/or fall pattern afterwards, with or without ST-T changes on ECG, in the absence of any other demonstrable cause for the chest pain. Unstable angina was diagnosed when, in the absence of the above troponin-I pattern, suggestive chest pain was associated with either transient ST-segment depression (≥ 0.1 mV) or T-wave inversion on ECG, pre-discharge ischemic stress test or significant coronary artery disease on angiography. Absence of ACS was diagnosed when complete diagnostic protocol was performed in the Chest Pain Unit and demonstrated neither myocardial necrosis nor ischemia. Patients who showed increased troponin-I levels after undergoing percutaneous coronary angioplasty were not considered to have myocardial infarction. TIMI risk score was calculated upon hospital admission and classified as low (0-2 points), intermediate (3-4 points) and high risk (5-7 points).

### Clinical endpoints

After hospital discharge, the patients' clinical status was prospectively determined by programmed telephone calls at 1 month, 1 year and 10 years. When any clinical event was reported by the patient or close relative, the hospital chart was reviewed or the private physician was contacted to establish the type of event or death. The primary endpoint of the study was all-cause death. At 10 years, 13 patients (5.5%) were lost to follow-up.

### Statistical analysis

All data analyses were performed using Stata Program 12.1 (StataCorp 2011). Plasma concentrations of BNP are described as median and interquartile (IQ) range. All sample measurements had their 95% confidence interval (CI) calculated according to the maximum likelihood estimation method. For all statistical analyses, a p-value ≤ 0.05 was considered significant.

Differences in proportions were assessed by using the chi-square method. The Mann-Whitney test was used to compare BNP levels between two independent groups (with or without 10-year all-cause death).

The bivariate analysis between BNP level quartiles and clinical variables and mortality was calculated by using the chi-square method for trend or the Fisher exact test.

Receiver operating characteristic (ROC) curves were generated and the area under the curve (and its 95% CI) was calculated to determine the best discriminating BNP level obtained on admission for predicting all-cause mortality.

Sensitivity, specificity, positive and negative predictive values, and 95% CI were calculated in the usual manner. Hazard ratio values were corrected when necessary to avoid calculation bias.

Cumulative 10-year survival was determined by the Kaplan-Meier method. Difference in survival according to BNP level was evaluated with a log-rank test and Cox proportional risk model.

Main effects logistic regression analyses were used to establish the predictive relationship between continuous or dichotomized BNP levels and mortality adjusted for the effects of clinical and laboratory variables. Initially, all significant variables identified on univariate analysis were included in the models. Variables were selected by use of the forward stepwise method guided by likelihood ratio, and respective c-statistics was calculated. Modeling was performed, refusing entry to variables with p > 0.10.

The increased discriminative value after addition of BNP to the established prognostic factors for 10-year mortality was estimated by using the integrated discrimination improvement (IDI) technique.^14^ IDI was calculated by analyzing the differences in patients' individual estimated probability of mortality after addition of BNP to a model containing the aforementioned established prognostic factors and represents the average improvement in predicted probability. Results were assessed by absolute and relative differences in discrimination values between models.

## Results

### Patients characteristics

From this cohort of 224 consecutive NSTEACS patients, 107 (47.8%) had a final diagnosis of myocardial infarction and 117 (52.2%), of unstable angina. Demographic and clinical characteristics are depicted in Table 1. TIMI risk score could be calculated in 202 of the 224 NSTEACS patients, most of whom were classified either as low or intermediate risk.

**Table 1:** Baseline clinical and laboratory data of 224 patients with non-ST elevation acute coronary syndrome

**Clinical characteristics**	**n = 224**
Age (years) (IQ range)	71.5 (60.5; 79)
Male gender	141 (62.9%)
Diabetes mellitus	53 (23.7%)
Smoking	36 (16.1%)
Previous infarct	69 (30.8%)
Previous use of aspirin	87 (38.8%)
Normal ECG (admission)	157 (70.1%)
ST-segment depression (admission)	28 (12.5%)
Left ventricular failure (admission)	26 (11.7%)
Low-risk TIMI score (TIMI 0-2)	76 (37.6%)
Intermediate-risk TIMI score (TIMI 3-4)	102 (50.5%)
High-risk TIMI score (TIMI 5-7)	24 (11.9%)
GFRe (mL/min) (IQ range)	69.3 (46.7; 92.3)
BNP (pg/mL) (admission) (IQ range)	81.9 (22.2; 225)
**Final diagnosis**	
Unstable angina	117 (52.2%)
NSTEMI	107 (47.8%)

IQ: interquartile; GFRe: estimated glomerular filtration rate; BNP: B-type natriuretic peptide; NSTEMI: non-ST elevation myocardial infarction; ECG: electrocardiogram

Table 2 demonstrates the relationship between BNP quartiles and clinical and laboratory findings in the 224 NSTEACS patients. It is noteworthy that there was a significant univariate direct relationship with admission BNP levels for most of these variables.

**Table 2:** Relationship of clinical and laboratory data, final diagnosis and all-cause mortality rate with quartiles of BNP levels (in pg/mL)

	**1st quartile**	**2nd quartile**	**3rd quartile**	**4th quartile**	
	**(BNP < 22.2) n = 56**	**(BNP 22.2-81.9) n = 56**	**(BNP 82.0-225) n = 56**	**(BNP > 225)n = 56**	**p-value**
Age (years) (IQ range)	60.5 (51.5; 71.5)	68.5 (58.5; 75)	73.0 (68; 81)	80.0 (73; 84)	p < 0.0001
Previous MI (%)	10 (17.9)	15 (26.8)	21 (37.5)	23 (41.1)	p = 0.0037
Previous use of aspirin (%)	16 (28.6)	23 (41.1)	29 (51.8)	19 (33.9)	p = 0.3594
Admission LV failure (%)	4 (7.3)	2 (3.6)	4 (7.1)	16 (28.6)	p = 0.0004
Admission ST-segment depression (%)	7.1 (4)	10.7 (6)	16.1 (9)	16.1 (9)	p = 0.1050
Admission TIMI Risk Score					
Low Risk (TIMI 0-2) (%)	31 (58.5)	19 (38.8)	11 (22.9)	15 (28.8)	
Intermediate Risk (TIMI 3-4) (%)	20 (37.7)	25 (51.0)	28 (58.3)	29 (55.8)	p = 0.0002
High Risk (TIMI 5-7) (%)	2 (3.8)	5 (10.2)	9 (18.8)	8 (15.4)	
LV dysfunction on Echo (%)	9 (16.4)	16 (30.2)	21 (38.2)	36 (64.3)	p < 0.0001
Final diagnosis					
Unstable angina (%)	60.7	64.3	55.4	28.6	p = 0.0004
NSTEMI (%)	39.3	35.7	44.6	71.4	p = 0.0004
All-cause death (%)	8 (14.3)	9 (16.1)	27 (48.2)	41 (73.2)	p < 0.0001

BNP- B: type natriuretic peptide; IQ: interquartile; MI: myocardial infarction; LV: left ventricle; Echo: echocardiogram; NSTEMI: non-ST elevation myocardial infarction.

During follow-up, 85 patients (37.9%) died in 10 years. Mortality rate increased progressively according to BNP quartiles levels, as depicted in Table 2. Patients who died were older at the index ACS event (79 *vs* 66 years, p < 0.001), had a worse mean estimated glomerular filtration rate (49.1 *vs* 80.5 mL/min, p < 0.001), presented more frequently with heart failure on admission (21.2 *vs* 5.8%, p = 0.001), had more frequently myocardial infarction on admission (61.2 *vs* 39.6%, p = 0.002) and had higher levels of admission BNP (220 *vs* 44.7 pg/mL, p < 0.001) than patients who survived.

### Prognostic accuracy of BNP levels measured on admission

ROC curve analysis disclosed 100 pg/mL as the best prognostic cut-off value of BNP for 10-year all-cause mortality (area under the curve = 0.789, 95% CI = 0.723 - 0.854). Patients with BNP ≥ 100 pg/mL had a mortality rate of 65% *vs* 17.3% of those with BNP < 100 pg/mL (relative risk = 3.76, p < 0.001). Sensitivity, specificity, and positive and negative predictive values for mortality were 74.1%, 75.5%, 64.9% and 82.7%, respectively.

The median 10-year survival, as determined by use of the Kaplan-Meier method, was 5.80 years (IQ range = 2.55-9.44) for patients with admission BNP ≥ 100 pg/mL *vs* 9.63 years (IQ range = 9.04-10.13) for those with BNP < 100 pg/mL (p < 0.0001).

On a multivariate stepwise logistic regression analysis adjusted for all demographic and clinical variables known to be predictors of cardiac death or related to an elevated BNP level (including all variables from Tables 1 and 2), a BNP level of 100 pg/mL obtained upon Emergency Department admission was an independent predictor of 10-year death (Table 3).

**Table 3:** Independent predictors of 10-year all-cause death by multivariate stepwise logistic regression analysis in patients with non-ST segment elevation acute coronary syndrome

**Independent predictors of mortality**		
**Variables**	**OR (95% CI)**	**p-value**
Age > 72 years	3.79 (1.62-8.86)	p = 0.002
BNP ≥ 100pg/mL	6.24 (2.95-13.23)	p < 0.001
GFRe (for each mL/min increment)	0.98 (0.97-0.99)	p = 0.049

BNP- B: type natriuretic peptide; GFRe: estimated glomerular filtration rate; OR: odds ratio; CI: confidence interval.

Finally, when using the IDI technique, the addition of BNP information to the traditional risk variables improved prediction and produced an absolute discriminatory increase rate of 3.06% for 10-year mortality (Table 4). The relative discrimination improvement was 10% greater for 10-year mortality with the knowledge and use of BNP information as compared to no use.

**Table 4:** Comparison of average risk and discrimination improvement using the traditional risk model and the B-type natriuretic peptide (BNP)-added model for 10-year mortality between patients who died (cases) and who survived (controls)

**10-year mortality**		**Average risk**	**Discrimination improvement**
Traditional risk model (without BNP)	Cases	24.931	34.557
	Controls	59.488	
BNP-added risk model	Cases	23.766	37.615
	Controls	61.381	

Integrated discrimination improvement: 37.615 – 34.557 = 3.058 (p = 0.0224) Relative discrimination improvement: 37.615 / 34.557 = 1.088

### TIMI risk score and BNP levels

**Figure 1:**
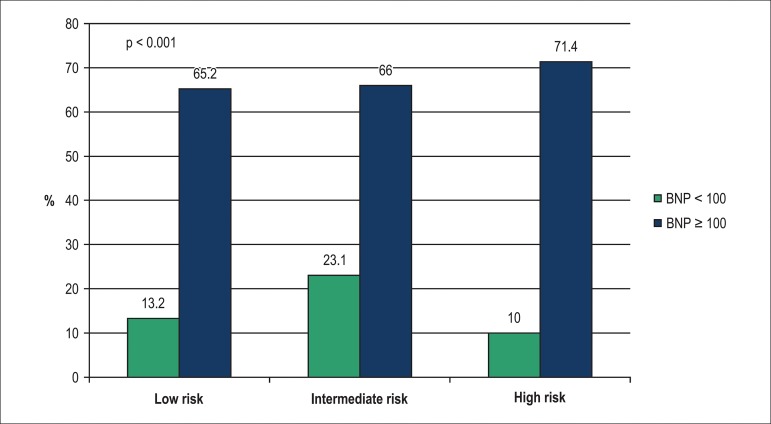
10-year all-cause mortality rates of non-ST segment elevation acute coronary syndrome patients according to the TIMI risk score levels (low = 0-2 points, intermediate = 3-4 points, high= 5-7 points) stratified by optimal C-statistics B-type natriuretic peptide (BNP) cut-off value (in pg/mL).

**Figure 2:**
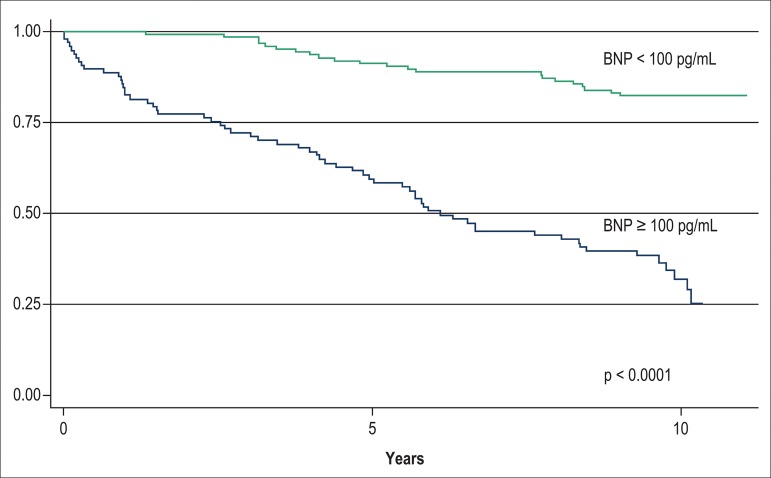
Kaplan-Meier survival curves of 224 patients with non-ST segment elevation acute coronary syndrome according to admission B-type natriuretic peptide (BNP) level.

**Figure 3:**
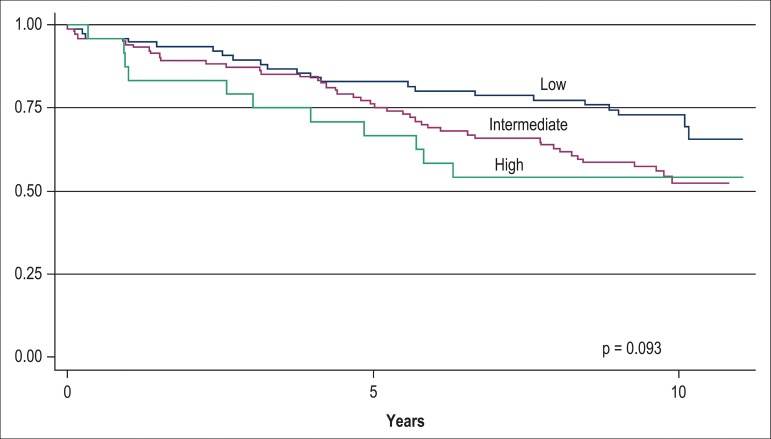
Kaplan-Meier survival curves of 202 patients with non-ST segment elevation acute coronary syndrome according to TIMI risk score levels (low = 0-2 points, intermediate = 3-4 points, high = 5-7 points).

Information of BNP level further improved risk stratification of the 10-year mortality rate in all three levels of the TIMI risk score, as depicted in Figure 1. The Kaplan-Meier 10-year survival curves of the two levels of BNP value and the three levels of TIMI risk score are depicted in Figures 2 and 3, and clearly disclose a much better discriminative prognostic performance of BNP.

## Discussion

Several cardiac biomarkers have been proposed and used in the last few decades for prognostic stratification of patients with ACS. The most used are the necrosis markers, specially troponins.^4,5,15,16^

Since the year 2000, the immediate- and short-term risk evaluation of cardiovascular outcomes in patients with ACS has been done with the TIMI^1^ and the GRACE^17^ risk scores as they are relatively easy to calculate at the bedside. It is important to remember that the TIMI risk score was originally tested for a 14-day follow-up; however, several authors have extrapolated its use for longer periods.^18-20^ Unfortunately these aggregates of risk factors lack good accuracy for 1-year follow-up as evaluated by the C-statistics of 0.58^18^ to 0.69^19^ for the TIMI and 0.71^18^ to 0.79^19^ for the GRACE risk scores. This knowledge has instigated the search for novel and more accurate markers of mortality prediction.^10,21-25^

BNP was initially found to be a very early and accurate biochemical marker of acute cardiac contractile dysfunction^26-28^ and has been also extensively studied in patients with myocardial ischemia and infarction.^29-31^ Its blood concentration increases immediately and rapidly after the beginning of cardiac hypoxia and correlates directly with the extension of myocardial ischemia.^11,32,33^ Lately, it has been found to be an excellent prognostic marker of cardiac outcomes in patients with ACS.^6,8,9,34-38^ However, most of these studies followed their patients for 1 year, and few of them did it for up to 4 years.^36,39-40^

The present study is the first to demonstrate that BNP measured on arrival at the Emergency Department is a strong and independent marker of all-cause death in patients with NSTEACS up to 10 years after the index event, even when compared to the TIMI Risk Score. Our population comes from a consecutive series of patients seen at the Emergency Department with acute chest pain to whom a systematic, careful, comprehensive diagnostic protocol for cardiac ischemia was applied, including the serial measurement of myocardial necrosis markers and ECG, as well as a single BNP sample obtained on admission. Patient's management was left at their private physicians discretion, but most of those with ACS were submitted to coronary angiography and revascularization if found necessary.

BNP was found to remain a powerful and independent marker of all-cause death in the long run in our NSTEACS study sample. BNP also had discriminatory power adding significant prognostic information beyond traditional risk variables for 10-year mortality as seen with the C-statistics. This was confirmed by use of the IDI tool that evaluates the absolute difference between predicted and observed outcome rates, thus representing predictive model's efficiency and consequently allowing comparison of two models. For 10-year mortality, IDI significantly improved outcome discrimination when BNP was used in the model as compared to not used (Table 4), allowing one more patient to obtain a correct outcome classification in a group of 33 individuals.

BNP added significant prognostic information to the TIMI Risk Score of these patients identifying lower and higher risk subgroups (Figure 1). Similar findings were previously demonstrated on a 6-month follow-up study by Bazzino et al.^6^ Contrary to BNP, the TIMI Risk Score itself could not accurately stratify patients for long-term mortality in our study (Figure 3).

Why an elevated BNP measured at an index ACS episode remains as a risk factor of all-cause mortality up to 10 years later is a matter of discussion. As BNP blood levels are not particularly predictive of recurrent fatal and nonfatal myocardial infarction,^6,41^ it seems unlikely that late mortality can be explained by a new episode of ACS. It may be speculated that its elevation represents a summation of two well-known prognostic markers in coronary artery disease, that is, the presence and extension of left ventricular ischemia and dysfunction.^7,42,43^ The findings of our study could be useful in treatment strategy decision as some authors have suggested that BNP-therapy-guided interventions might improve mortality after ACS.^44,45^

### Limitations of the study

The present study sample is relatively small in comparison with other cited multicenter studies and originates from a single, private, cardiology-oriented institution. This study does not address the possible value of serial BNP measurements. The troponin assay used in this patient sample is of an older generation (non-high sensitive), although many institutions around the world still use it. It must be remembered that the biochemical diagnostic criteria of myocardial infarction have changed at least twice in the last decade.^46,47^

## Conclusions

BNP measured on hospital admission in patients with NSTEACS remains a strong, independent predictor of very long-term all-cause mortality, corroborating its excellent risk-stratification capability seen in short- and intermediate-term follow-up studies. This study allows raising the hypothesis that BNP should be measured in all patients with NSTEACS at the index event for long-term risk stratification.
